# Targeting senescent cells alleviates obesity‐induced metabolic dysfunction

**DOI:** 10.1111/acel.12950

**Published:** 2019-03-25

**Authors:** Allyson K. Palmer, Ming Xu, Yi Zhu, Tamar Pirtskhalava, Megan M. Weivoda, Christine M. Hachfeld, Larissa G. Prata, Theo H. van Dijk, Esther Verkade, Grace Casaclang‐Verzosa, Kurt O. Johnson, Hajrunisa Cubro, Ewald J. Doornebal, Mikolaj Ogrodnik, Diana Jurk, Michael D. Jensen, Eduardo N. Chini, Jordan D. Miller, Aleksey Matveyenko, Michael B. Stout, Marissa J. Schafer, Thomas A. White, LaTonya J. Hickson, Marco Demaria, Vesna Garovic, Joseph Grande, Edgar A. Arriaga, Folkert Kuipers, Thomas von Zglinicki, Nathan K. LeBrasseur, Judith Campisi, Tamar Tchkonia, James L. Kirkland

**Affiliations:** ^1^ Robert and Arlene Kogod Center on Aging Mayo Clinic Rochester Minnesota; ^2^ Medical Scientist Training Program Mayo Clinic Rochester Minnesota; ^3^ Department of Internal Medicine Mayo Clinic Rochester Minnesota; ^4^ University of Connecticut Center on Aging, University of Connecticut Health Farmington Connecticut; ^5^ Department of Laboratory Medicine University Medical Center Groningen, University of Groningen Groningen The Netherlands; ^6^ Department of Pediatrics University Medical Center Groningen, University of Groningen Groningen The Netherlands; ^7^ Division of Nephrology and Hypertension Mayo Clinic Rochester Minnesota; ^8^ Institute for Ageing, Ageing Research Laboratories Newcastle University Newcastle upon Tyne UK; ^9^ Department of Physiology and Biomedical Engineering Mayo Clinic Rochester Minnesota; ^10^ Department of Anesthesiology Mayo Clinic Rochester Minnesota; ^11^ Department of Surgery Mayo Clinic Rochester Minnesota; ^12^ Department of Nutritional Sciences University of Oklahoma Health Sciences Center Oklahoma City Oklahoma; ^13^ Reynolds Oklahoma Center on Aging University of Oklahoma Health Sciences Center Oklahoma City Oklahoma; ^14^ Harold Hamm Diabetes Center University of Oklahoma Health Sciences Center Oklahoma City Oklahoma; ^15^ Division of Geriatric Medicine and Gerontology Mayo Clinic Rochester Minnesota; ^16^ Buck Institute for Research on Aging Novato California; ^17^ European Research Institute for the Biology of Ageing University Medical Center Groningen, University of Groningen Groningen The Netherlands; ^18^ Department of Pathology Mayo Clinic Rochester Minnesota; ^19^ Department of Chemistry University of Minnesota Minneapolis Minnesota; ^20^ Department of Physical Medicine and Rehabilitation Mayo Clinic Rochester Minnesota

**Keywords:** adipogenesis, aging, cellular senescence, dasatinib, quercetin, senolytics, type 2 diabetes

## Abstract

Adipose tissue inflammation and dysfunction are associated with obesity‐related insulin resistance and diabetes, but mechanisms underlying this relationship are unclear. Although senescent cells accumulate in adipose tissue of obese humans and rodents, a direct pathogenic role for these cells in the development of diabetes remains to be demonstrated. Here, we show that reducing senescent cell burden in obese mice, either by activating drug‐inducible “suicide” genes driven by the p16*^Ink4a^* promoter or by treatment with senolytic agents, alleviates metabolic and adipose tissue dysfunction. These senolytic interventions improved glucose tolerance, enhanced insulin sensitivity, lowered circulating inflammatory mediators, and promoted adipogenesis in obese mice. Elimination of senescent cells also prevented the migration of transplanted monocytes into intra‐abdominal adipose tissue and reduced the number of macrophages in this tissue. In addition, microalbuminuria, renal podocyte function, and cardiac diastolic function improved with senolytic therapy. Our results implicate cellular senescence as a causal factor in obesity‐related inflammation and metabolic derangements and show that emerging senolytic agents hold promise for treating obesity‐related metabolic dysfunction and its complications.

## 1 INTRODUCTION

The prevalence of type 2 diabetes has quadrupled since 1980 (World Health Organization, 2016). It is associated with multi‐organ complications, including cardiovascular and renal disease. Obesity and, more specifically, dysfunctional adipose tissue are strongly associated with whole‐body insulin resistance and type 2 diabetes mellitus (Kahn & Flier, 2000; Palmer et al., 2015). Senescent cells accumulate in adipose tissue of obese and diabetic humans and mice (Minamino et al., 2009; Schafer et al., 2016; Tchkonia et al., 2010), but it is unclear whether they are merely associated with diabetes or if their presence is a causal driver.

Cellular senescence is a cell fate that entails proliferative arrest and acquisition of a pro‐inflammatory senescence‐associated secretory phenotype (SASP; Coppe et al., 2008). Although senescent cells exist in relatively small numbers in any particular tissue, they have been associated with multiple diseases of aging and are emerging as useful therapeutic targets for age‐related diseases, including cardiovascular disease, pulmonary fibrosis, neurodegeneration, and osteoporosis (Farr et al., 2017; Musi et al., 2018; Roos et al., 2016; Schafer et al., 2017). A number of stimuli, including potentially oncogenic, inflammatory, damage‐related, and metabolic stimuli, can trigger a senescence response (Munoz‐Espin & Serrano, 2014). Components of the SASP secreted by adipose‐derived senescent cells have been postulated to confer insulin resistance upon metabolic tissues, inhibit adipogenesis, and attract immune cells that can exacerbate insulin resistance (Xu, Palmer et al., 2015). Here, we determined whether removing senescent cells in the context of obesity improves metabolic phenotypes.

Recently, drugs that preferentially decrease senescent cell burden, termed senolytics, have been identified (Kirkland, Tchkonia, Zhu, Niedernhofer, & Robbins, 2017). We discovered the first senolytics based on our observation that senescent cells rely on several survival pathways, including those regulated by PI3K/AKT‐, p53/p21/serpine‐, HIF‐1α‐, and BCL‐2/BCL‐X_L_‐family components, to confer resistance to their pro‐apoptotic SASP and intracellular cell damage signals (Zhu et al., 2016,2015). Knowing this, we identified dasatinib (D) and quercetin (Q) as orally bioactive drugs that transiently target these survival pathways to induce apoptosis preferentially in senescent cells (Zhu et al., 2015). Subsequently, we and others found that navitoclax, (ABT263, which targets Bcl‐xL, Bcl‐2, and Bcl‐w but not Mcl‐1), is also senolytic (Chang et al., 2016; Zhu et al., 2016). We showed that D and Q, alone and in combination, cause apoptosis in senescent cells without significant effects in quiescent or proliferating cells (Xu et al., 2018; Zhu et al., 2015). Senolytics do not prevent the generation of senescent cells and they are effective when administered intermittently, which could help to mitigate any potential negative effects of senescent cell removal, such as delayed wound healing (Demaria et al., 2017; Zhu et al., 2015).

We employed the combination of D plus Q (D + Q) in our studies for the following reasons. (a) In our hands, no senolytic investigated thus far targets all types of senescent cells (Kirkland & Tchkonia, 2017). For example, unlike navitoclax (ABT263), fisetin, A1331852, A1155463 (Zhu et al., 2017,2016,2015), or Q on its own, D selectively targets senescent adipose progenitors (Zhu et al., 2015), a key cell type for adipose tissue and metabolic function (Tchkonia et al., 2013). (b) On the other hand, Q, unlike D, is effective against senescent endothelial cells (Zhu et al., 2015), a cell type implicated in vascular complications of diabetes (Caballero, 2003). (c) D + Q is effective in alleviating multiple age‐ and senescence‐associated disorders, including many that are frequent complications or comorbidities of diabetes in preclinical animal models; these comorbidities include arteriosclerosis, vascular hyporeactivity, osteoporosis, hepatic steatosis, physical dysfunction, neurodegeneration, and neuropsychiatric dysfunction (Farr et al., 2017; Kirkland & Tchkonia, 2017; Kirkland et al., 2017; Musi et al., 2018; Ogrodnik et al., 2017,2019; Roos et al., 2016; Xu et al., 2018). (d) Navitoclax and other BCL‐2 family member inhibitors can be toxic, for example, causing severe thrombocytopenia, which can occur even with intermittent dosing (Wilson et al., 2010). Navitoclax can also cause neutropenia, complicating interpretation of whether its effects are due to senolytic activity or immune system suppression. For these reasons, we elected to focus on D + Q.

Here, we characterize the effects of eliminating senescent cells on obesity‐induced derangements in adipose tissue function and glucose homeostasis. To do this, we used both transgenic mouse models and treatment with the senolytics, D + Q. Our findings support the idea that senescent cells could be a novel therapeutic target for treating obesity‐induced metabolic dysfunction.

## 2 RESULTS

### 2.1 Senescent cells accumulate in visceral fat in obesity

We used two transgenic mouse models from which senescent cells can be selectively cleared: (a) p16‐3MR mice, in which a long *p16^Ink4a^*‐promoter sequence drives expression of a trimodal reporter‐killer fusion protein (3MR), allowing senescent cell killing by ganciclovir (GCV) and identification by whole‐body luminescence (Demaria et al., 2014) and (b) INK‐ATTAC mice, in which a truncated *p16^Ink4a^*‐promoter sequence drives expression of a vFKBP‐caspase‐8‐FLAG fusion protein that can be activated by AP20187, a vFKBP dimerizer, to cause senescent cell apoptosis (Baker et al., 2011). Cells that highly express p16^Ink4a^ are targeted in the INK‐ATTAC and p16‐3MR models. However, not all cells with high p16^Ink4a^ expression are senescent, and not every senescent cell highly expresses p16^Ink4a^ (Hall et al., 2017; Okuma, Hanyu, Watanabe, & Hara, 2017), limiting the sensitivity and specificity for senescent cell killing in these mouse models. Therefore, we used an additional distinct, potentially translatable approach for eliminating senescent cells, that is, senolytic drugs, which target anti‐apoptotic pathways in senescent cells and do not depend on p16^Ink4a^ expression to reduce senescent cell burden (Kirkland et al., 2017; Zhu et al., 2015).

**Figure 1:**
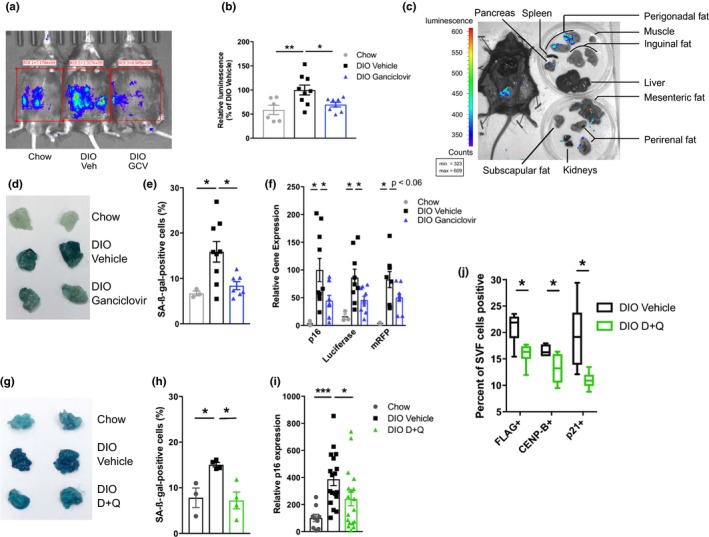
Removal of obesity‐induced senescent cells from adipose tissue. (a, b) *Renilla* luciferase activity in DIO p16‐3MR mice (a, representative image), quantified in b (*n* = 6–9 per group). (c) *Renilla* luciferase activity localization in DIO p16‐3MR dissected tissues. (d, e) Senescence‐associated beta‐galactosidase (SA‐β‐gal) activity as whole tissue activity (d; representative image) and % positive cells of total DAPI^+^ cells in p16‐3MR VAT (e; chow *n* = 3, DIO *n* = 7–9 per group). (f) Expression of p16‐3MR transgene (eGFP) components (*Renilla* luciferase and mRFP) and p16^Ink4a^ (chow *n* = 3, DIO *n* = 11 per group) in p16‐3MR VAT. (g, h) Senescence‐associated beta‐galactosidase (SA‐β‐gal) activity as whole tissue activity (g; representative image) and % positive cells of total DAPI^+^ cells in p16‐3MR VAT (h; *n* = 3–4 per group). (i) p16^Ink4a^ mRNA levels (c; chow *n* = 8, DIO *n* = 19–21 per group) in VAT of D + Q‐treated DIO mice. (j) Percent of VAT stromal vascular fraction (SVF) cells highly expressing FLAG (a component of the *p16^Ink4a^* promoter‐driven ATTAC fusion protein), CENP‐B, and p21^Cip1^ after a single course of D + Q (o, *n* = 6 per group) in DIO INK‐ATTAC mice. Means ± *SEM* are shown. Box and whisker plot show minimum, mean, maximum, 25th and 75th percentiles. *p < 0.05, **p < 0.005, ***p < 0.0005; one‐way ANOVA with Bonferroni correction or two‐tailed Student's *t* test when comparing two groups

Obesity was induced either by high‐fat diet (diet‐induced obesity, DIO, with ad libitum chow‐fed controls) or by genetic means in leptin receptor knockout (*db/db*) mice (Supporting Information Figure S1). Senescent cell abundance, as measured by luciferase activity in p16‐3MR mice, increased in response to DIO compared to chow‐fed mice (Figure 1a–c and Supporting Information Figure S2a,b). Bioluminescent imaging (BLI) in ex vivo tissues showed that the most prominent signal was present in visceral (specifically perigonadal) adipose tissue (VAT; Figure 1c and Supporting Information Figure S2b). Although senescent cells are known to accumulate in other organs including the liver after high‐fat feeding (Ogrodnik et al., 2017; Yoshimoto et al., 2013), senescent cell abundance detectible in excised tissues by BLI was not as pronounced in other adipose tissue depots, skeletal muscle, liver, and pancreas (Figure 1c). That said, limitations of BLI, including a high threshold to visualize senescent cells and limited tissue penetration of the *Renilla *luciferase signal, could have contributed to our inability to detect bioluminescence in these other tissues. Therefore, we used additional, more sensitive methods including gene expression, senescence‐associated beta‐galactosidase staining, and mass cytometry to assess senescent cell abundance in the studies reported below.

### 2.2 Senescent cell clearance improves glucose homeostasis and insulin sensitivity

Senescent cell abundance declined after intermittent administration of ganciclovir to p16‐3MR DIO mice (Figure 1a,b,d–f,), AP20187 to DIO INK‐ATTAC mice (Supporting Information Figure S2c,d) and INK‐ATTAC; *db/db* mice (Supporting Information Figure S2e), and D + Q to wild‐type DIO mice (Figure 1g–i). CyTOF analyses conducted after a single course of AP20187 or D + Q in INK‐ATTAC mice showed that adipose tissue senescent cells highly expressing *p16^Ink4a^*‐promoter‐induced FLAG, CENP‐B, or p21^Cip1^ were decreased significantly (Figure 1j and Supporting Information Figure S2f). In these experiments, adipose progenitor cells, rather than endothelial cells, macrophages, or T cells, were the main cell type targeted (Supporting Information Figure S2g,h).

**Figure 2:**
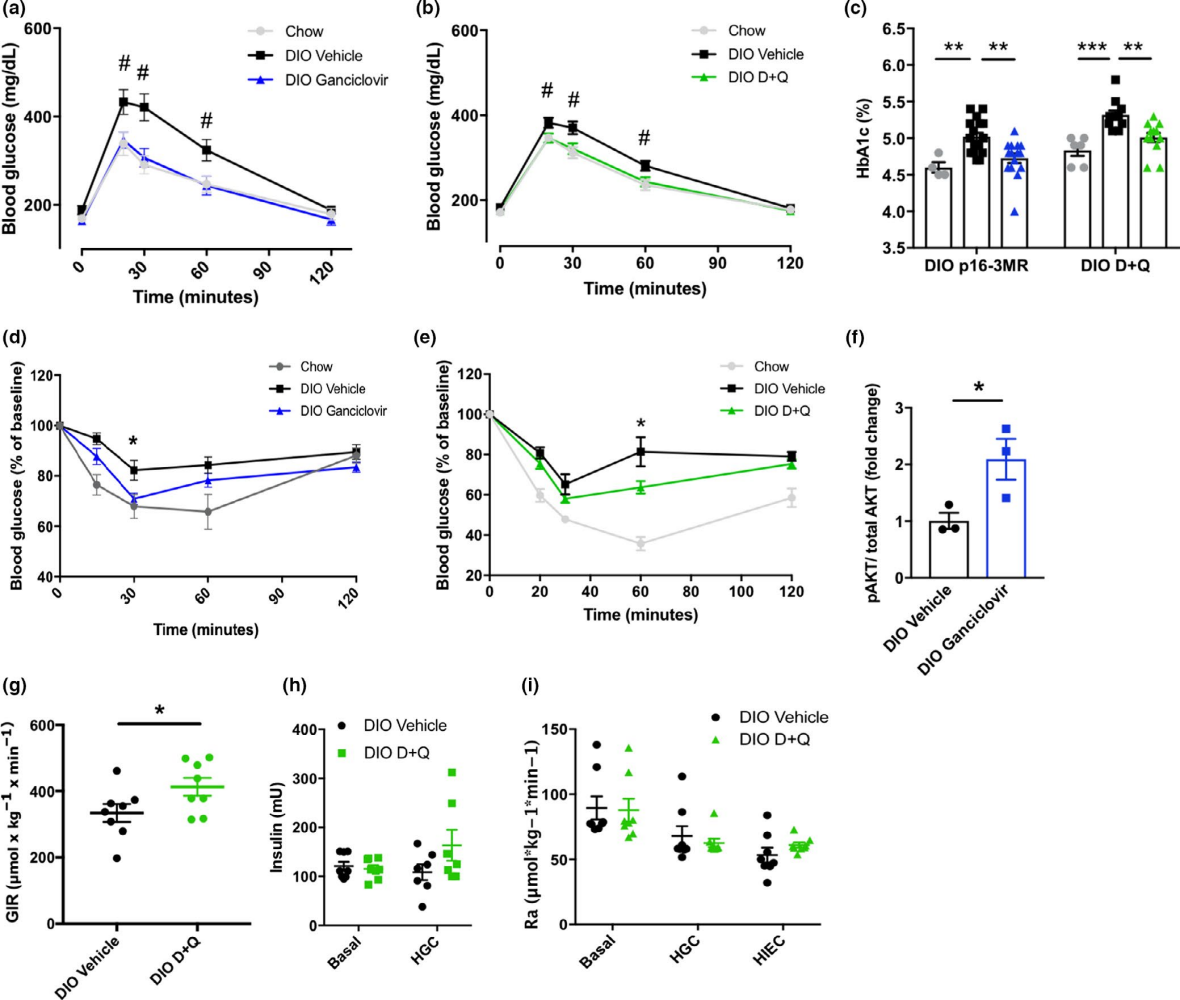
Eliminating senescent cells enhances glucose homeostasis and insulin sensitivity. (a, b) Intraperitoneal glucose tolerance test in DIO p16‐3MR (a; chow *n* = 4, DIO *n* = 6–7 per group) and DIO wild‐type mice treated with D + Q (b, chow *n* = 4, DIO *n* = 11 per group) following senescent cell clearance. (c) Hemoglobin A1c in DIO p16‐3MR (chow *n* = 4, DIO *n* = 15–18 per group) and DIO wild‐type mice treated with D + Q (chow *n* = 6, DIO *n* = 11–12 per group). (d, e) ITT following ganciclovir treatment in p16‐3MR mice (d, *n* = 4 chow, *n* = 18–19 DIO groups), or D + Q treatment in DIO wild‐type mice (e, chow *n* = 6, DIO *n* = 11–12 per group). (f) Fold change in AKT serine‐473 phosphorylation after 5‐min ex vivo 5 nM insulin stimulation in freshly isolated p16‐3MR VAT (*n* = 3 per group). For each mouse, p‐AKT was normalized to total AKT and expressed as a ratio of p‐AKT in insulin‐exposed tissue to p‐AKT in noninsulin‐exposed tissue. (g) Glucose infusion rate (GIR) during hyperinsulinemic–euglycemic clamp in DIO mice treated with vehicle or D + Q (*n* = 8 per group). (h) Plasma insulin concentration at baseline and during hyperglycemic clamping (HGC) in DIO mice treated with D + Q (*n* = 8 per group). (i) Glucose appearance rate (Ra) during basal, hyperglycemic, and hyperinsulinemic–euglycemic clamping in DIO mice treated with D + Q (*n* = 8 per group). Means ± *SEM* are shown. *p < 0.05, **p < 0.005, ***p < 0.0005; one‐way ANOVA with Bonferroni correction for multiple comparisons. ^#^ *p* < 0.05, two‐tailed Student's *t* test comparing DIO vehicle‐treated group to DIO ganciclovir‐treated, or D + Q‐treated group. Groups of interest were compared at each time point for GTTs and ITTs

Clearing senescent cells improved glucose tolerance (Figure 2a,b and Supporting Information Figure S3a,b) and reduced hemoglobin A1c (HbA1c), a marker of long‐term glucose control (Figure 2c and Supporting Information Figure S3c). These effects were not seen in lean, chow‐fed control mice (Supporting Information Figure S3d–g), wild‐type (i.e., not p16‐3MR) DIO mice treated with ganciclovir (Supporting Information Figure S3h,i), or DIO mice treated with navitoclax, which does not target senescent adipocyte progenitors (Zhu et al., 2016) (Supporting Information Figure S3j). The time course of metabolic improvement following initiation of D + Q treatment paralleled that of clearance of high p16^Ink4a^‐expressing cells by engaging death mechanisms due to transgenes (Supporting Information Figure S3k,l). Eliminating senescent cells did not affect body weight, activity, or food intake, consistent with improved glucose homeostasis being due principally to increased insulin sensitivity (Supporting Information Figure S3m–r).

After senescent cell reduction, DIO mice became more insulin sensitive, as indicated by insulin tolerance testing (ITT; Figure 2d,e and Supporting Information Figure S4a) and an increased glucose infusion rate during hyperinsulinemic clamping (Figure 2g). Furthermore, AKT Ser473 phosphorylation increased in response to ex vivo insulin stimulation of adipose tissue freshly harvested from animals that had undergone senescent cell clearance (Figure 2f and Supporting Information Figure S4b). The insulin‐positive pancreatic islet area remained unchanged after depleting senescent cells (Supporting Information Figure S4c–e). Plasma insulin concentrations were lower in response to a glucose challenge in both p16‐3MR mice treated with ganciclovir and DIO mice treated with D + Q (Supporting Information Figure S4f,g). Pancreatic insulin secretion was unchanged in D + Q‐treated mice during hyperglycemic clamping experiments (Figure 2h). Insulin sensitivity was not affected by genetic interventions that cause elimination of senescent cells in age‐matched lean mice (Supporting Information Figure S4h) or obese WT mice (Supporting Information Figure S4i), indicating that off‐target effects of AP20187 or ganciclovir are unlikely to have contributed substantially to the observed metabolic improvements. These results indicate that, at least using these methods for senescent cell ablation in mice with DIO‐induced metabolic dysfunction, metabolic benefits were primarily due to improved peripheral insulin sensitivity rather than β‐cell compensation. Hepatic glucose production was also found to be unchanged upon D + Q treatment in DIO mice under basal, hyperglycemic, and hyperinsulinemic conditions (Figure 2i). Our findings do not preclude the possibility that other approaches for clearing senescent cells or at different stages during development of obesity‐induced metabolic dysfunction might affect hepatic glucose production or pancreatic insulin secretion.

### 2.3 Adipogenic potential is improved after senescent cell reduction

Circulating and adipose tissue inflammatory mediators, some of which are components of the SASP (Coppe et al., 2008; Tchkonia et al., 2010), increase in obesity and can impede adipogenesis and contribute to insulin resistance (Kahn & Flier, 2000; Xu, Tchkonia et al., 2015). In p16‐3MR mice but not WT mice treated with ganciclovir, plasma IFN‐γ and IL‐1β concentrations were decreased (Supporting Information Figure S5a,b). Cells expressing high concentrations of TNF‐α isolated from the visceral adipose tissue (VAT) stromal vascular fraction declined upon AP20187 treatment of DIO INK‐ATTAC mice (Supporting Information Figure S5c). Plasma adiponectin, which is associated with improved insulin sensitivity (Kadowaki et al., 2006), also increased after senescent cell reduction in p16‐3MR mice (Supporting Information Figure S5d,e), and adipose tissue IFN‐γ expression was reduced (Supporting Information Figure S5f).

**Figure 3:**
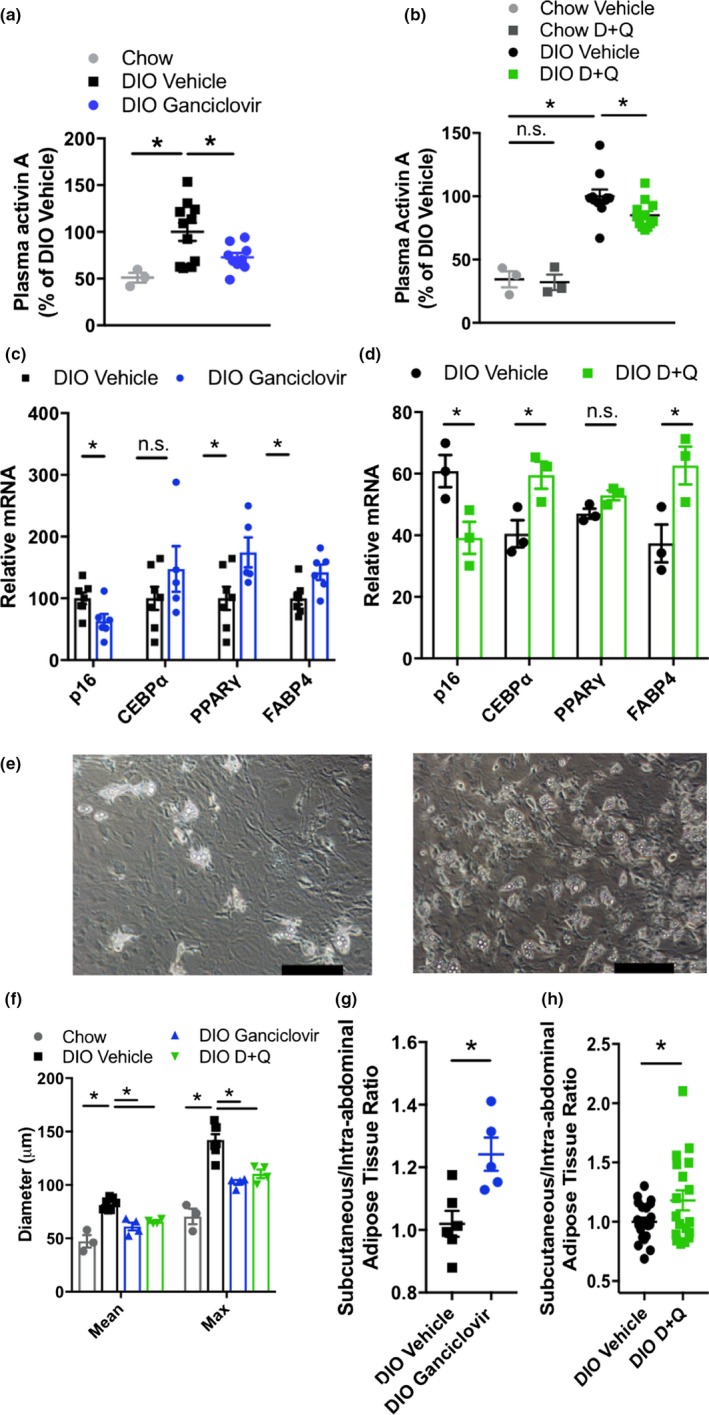
Adipogenesis is enhanced by senescent cell reduction. (a, b) Plasma activin A in p16‐3MR mice (a; chow *n* = 3, DIO *n* = 9–11) and D + Q‐treated DIO mice (b, chow *n* = 3, DIO *n* = 11–12). (c, d) Adipogenic gene expression in cells isolated from subcutaneous adipose tissue stromal vascular fraction (SVF) of p16‐3MR (c; *n* = 5–7 per group) and D + Q‐treated mice (d, *n* = 3 per group). (e) Representative images of lipid droplet formation during differentiation of adipocyte progenitors isolated from vehicle‐ or ganciclovir‐treated p16‐3MR mice after 5‐day exposure to differentiation medium (scale bars indicate 50 μm). (f) VAT cell size in p16‐3MR mice and D + Q‐treated mice (*n* = 3–7 per group). (g, h) Subcutaneous:intra‐abdominal adipose ratio in DIO p16‐3MR mice (g, *n* = 5–6 per group) and D + Q‐treated mice (h, *n* = 18–21 per group). Means ± *SEM* are shown. *p < 0.05; one‐way ANOVA with Bonferroni correction or two‐tailed Student's *t* test when comparing two groups

Activin A, a TGF‐β/GDF‐related protein that is a component of the senescent adipocyte progenitor SASP (Xu, Palmer et al., 2015), was increased in DIO mice (Figure 3a,b) and correlated with highly p16^Ink4a^‐expressing senescent cell burden, as manifested by *p16^Ink4a^*‐promoter‐driven luciferase expression in p16‐3MR mice (Supporting Information Figure S5g). Activin A impedes expression of the insulin‐sensitizing adipogenic transcription factors PPARγ and C/EBPα, thereby contributing to insulin resistance (Hamm, Jack, Pilch, & Farmer, 1999; Xu, Palmer et al., 2015; Zaragosi et al., 2010). Senescent cell ablation abrogated the DIO‐induced increase in activin A (Figure 3a,b). This was associated with higher expression of adipogenic transcription factors and their targets in adipocyte progenitors from obese mice (Figure 3c,d) and enhanced adipogenic differentiation in culture (Figure 3e). These findings are consistent with previous data showing that senescent adipocyte progenitors develop a SASP that inhibits adipogenesis (Xu, Palmer et al., 2015). It was also previously found that BrdU incorporation increases in adipose tissue following AP20187 treatment in INK‐ATTAC mice (Baker et al., 2011), suggesting that cleared senescent cells can be replaced by nonsenescent, proliferation‐competent adipocyte progenitors that can then differentiate into insulin‐responsive fat cells.

We found that senescent cell clearance decreases adipocyte hypertrophy (Figure 3f and Supporting Information Figure S5h) and increases the ratio of subcutaneous to intra‐abdominal adipose tissue (Figure 3g,h), reflective of better insulin sensitivity (Gustafson, Hedjazifar, Gogg, Hammarstedt, & Smith, 2015). These changes in adipose tissue distribution were mainly due to expansion of subcutaneous depots (Supporting Information Figure S5i,j). Furthermore, DIO INK‐ATTAC mice treated with AP20187 had fewer lipid droplets in muscle, measured by oil red O staining (Supporting Information Figure S5k), and less severe hepatic steatosis (Ogrodnik et al., 2017). Collectively, these findings indicate that decreasing the burden of senescent cells may enhance insulin sensitivity in part by improving the proliferative and differentiation potential of adipocyte progenitors, contributing to healthier adipose tissue distribution and limiting ectopic lipid deposition (Gustafson et al., 2015).

### 2.4 Macrophage abundance is correlated with senescent cell burden in adipose tissue

**Figure 4:**
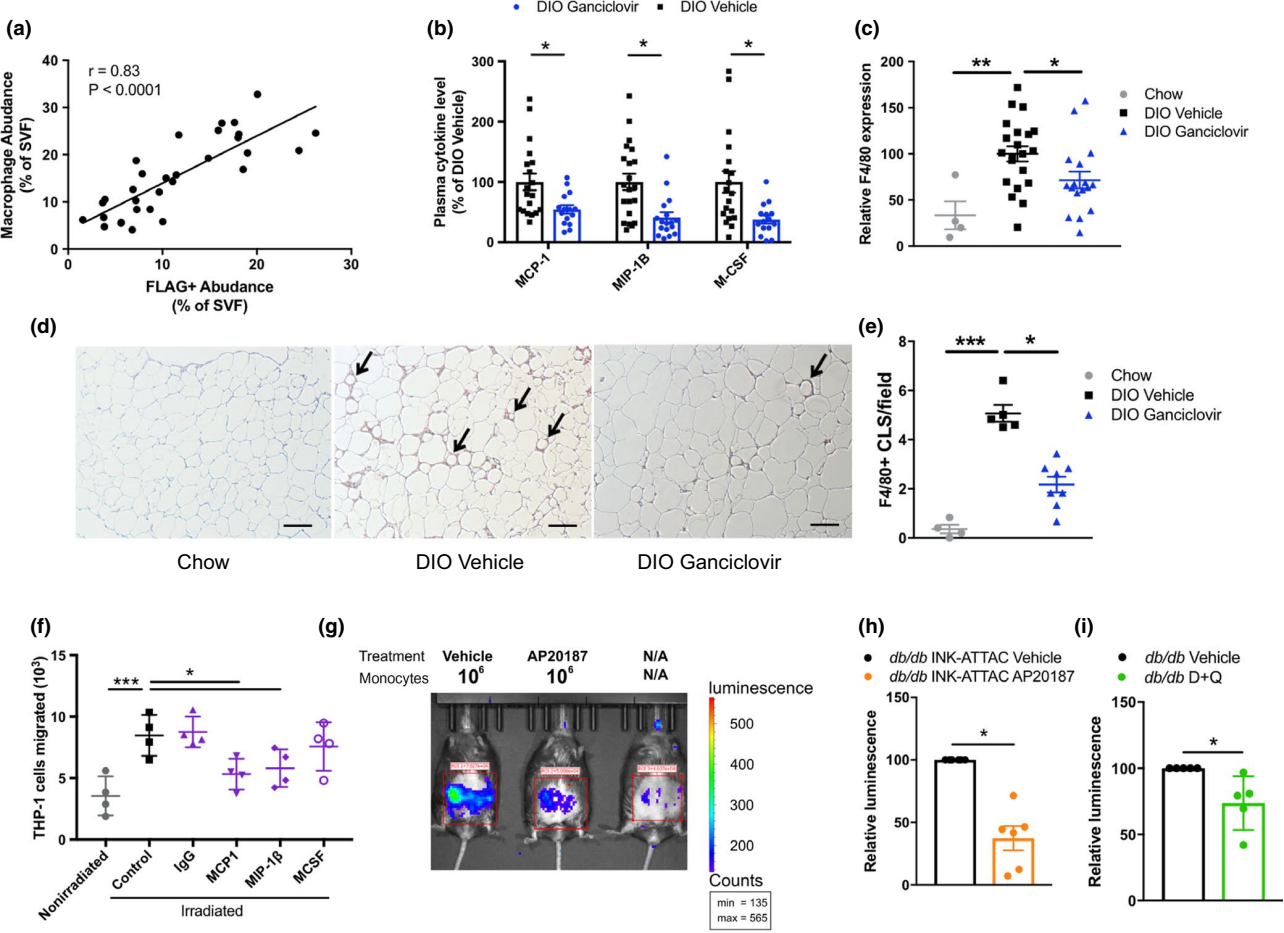
Senescent cells promote macrophage infiltration. (a) Correlation of FLAG^+^ cells with F4/80^+^/Cd11b^+^ macrophages in the SVF of DIO INK‐ATTAC VAT as analyzed by CyTOF (*n* = 30). (b) Plasma cytokines in ganciclovir‐treated DIO p16‐3MR mice (*n* = 19–22 per group). (c) F4/80 mRNA in VAT of ganciclovir‐treated p16‐3MR DIO mice (chow *n* = 4, DIO *n* = 17–22 per group). (d, e) F4/80^+^ crown‐like structures in VAT of ganciclovir‐treated p16‐3MR DIO mice (representative photographs in d, quantified in e; chow *n* = 4; DIO *n* = 5–8 per group). (f) THP‐1 cell migration into a Transwell containing either nonirradiated or irradiated (senescent) adipocyte progenitors, with no addition of antibody (control), addition of nonspecific antibody (IgG), or addition of antibodies against MCP‐1, MIP1‐β, or M‐CSF (*n* = 4 per condition). (g, h) Representative image of luminescence signal in AP20187‐treated INK‐ATTAC;*db/db* mice 24 hr following i.v. injection of 1 × 10^6^ luciferase^+^ monocytes (g), normalized to vehicle‐treated mice and quantified in h (*n* = 6 per group). (i) Quantification of luminescence in D + Q‐treated *db/db* mice 24 hr following i.v. injection of 1 × 10^6^ luciferase^+^ monocytes isolated from CAG‐luc mice, normalized to vehicle‐treated mice (*n* = 5 per group). Means ± *SEM* are shown. *p < 0.05, **p < 0.005, ***p < 0.0005; one‐way ANOVA with Bonferroni correction or two‐tailed Student's *t* test when comparing two groups

Macrophages, activated CD4^+^ lymphocytes, and other immune cells accumulate in adipose tissue of obese individuals (Olefsky & Glass, 2010; Tesch, 2007). Like senescent cells, these immune cells release inflammatory mediators, including TNF‐α and osteopontin, which can contribute to obesity‐related insulin resistance (Tardelli et al., 2016; Tesch, 2007). Events responsible for obesity‐related immune cell infiltration have remained largely elusive. In VAT of DIO mice, macrophage abundance correlated with highly p16^Ink4a^‐expressing senescent cells (as measured by *p16^Ink4a^*‐promoter‐induced FLAG) in vivo (Figure 4a).

Treatment with ganciclovir in DIO p16‐3MR but not DIO wild‐type mice reduced plasma concentrations of the macrophage‐attracting chemokines, MCP‐1 and MIP‐1β, as well as macrophage‐colony‐stimulating factor (M‐CSF) (Figure 4b and Supporting Information Figure S6a). Expression of the macrophage marker, F4/80, significantly declined in adipose tissue after 2–3 months of intermittent treatment to remove senescent cells (Figure 4c and Supporting Information Figure S6b), and fewer crown‐like structures were found in perigonadal adipose tissue (Figure 4d,e). Also, factors secreted by adipose tissue activated T cells, including IL‐17 (Supporting Information Figure S6c,d) and osteopontin (Supporting Information Figure S6e–g; Tardelli et al., 2016; Zuniga et al., 2010), which is also a component of the adipose progenitor SASP in humans (Supporting Information Figure S6h), declined after reducing senescent cell burden.

Activated macrophage contamination is a potentially significant confounder in these studies, since macrophages can express p16^Ink4a^, β‐galactosidase, and SASP‐like factors (Hall et al., 2017). Unlike truly senescent cells, macrophages can revert to a nonactivated state, potentially even into monocytes that replicate. Therefore, we conducted CyTOF studies and specifically assayed the macrophage markers, F4/80 and Cdllb, and verified that AP20187 and D + Q do not specifically and immediately target activated macrophages, as this population did not decrease after one round of treatment with either agent (Supporting Information Figure S2g,h). In addition, we have previously shown that D + Q treatment sufficient to reduce senescent preadipocyte abundance does not directly reduce macrophage abundance in freshly isolated adipose tissue explants from obese human subjects (see Supporting Information Figure S9 in Xu et al., 2018).

### 2.5 Senescent cell clearance decreases macrophage homing to adipose tissue

We found that senescent adipocyte progenitors attract monocytes in culture (Xu, Tchkonia et al., 2015; Supporting Information Figure S6i) and that this effect is mitigated by the addition of neutralizing antibodies against the SASP components MCP‐1 and MIP‐1β, but not M‐CSF (Figure 4f). To test whether senescent cells can directly cause immune cell infiltration and therefore whether targeting senescent cells reduces subsequent macrophage burden in adipose tissue, we injected monocytes that constitutively express luciferase intravenously into lean and obese (*db/db*) nonluciferase‐expressing INK‐ATTAC or wild‐type mice. We used *db/db* mice in these experiments due to their highly inflammatory adipose tissue phenotype. The injected cells homed mainly to the VAT of obese mice, where obesity‐induced senescent cells concentrate, with little to no luminescence detected in lean mice (Supporting Information Figure S6j–l). Luciferase‐expressing monocytes injected into obese INK‐ATTAC;*db/db* mice 4 days after AP20187 treatment, or into wild‐type *db/db* mice 4 days after D + Q treatment, exhibited decreased migration into VAT compared to vehicle‐treated mice (Supporting Information Figure 4g–i). AP20187 had no evident effect on monocyte migration in control *db/db *mice without the INK‐ATTAC transgene (Supporting Information Figure S46m,n). Thus, senescent cells can cause macrophage migration into adipose tissue in obesity, and targeting senescent cells prevents and reduces the adipose tissue macrophage accumulation that is often associated with obesity.

### 2.6 Decreasing senescent cell burden may alleviate complications of diabetes

**Figure 5:**
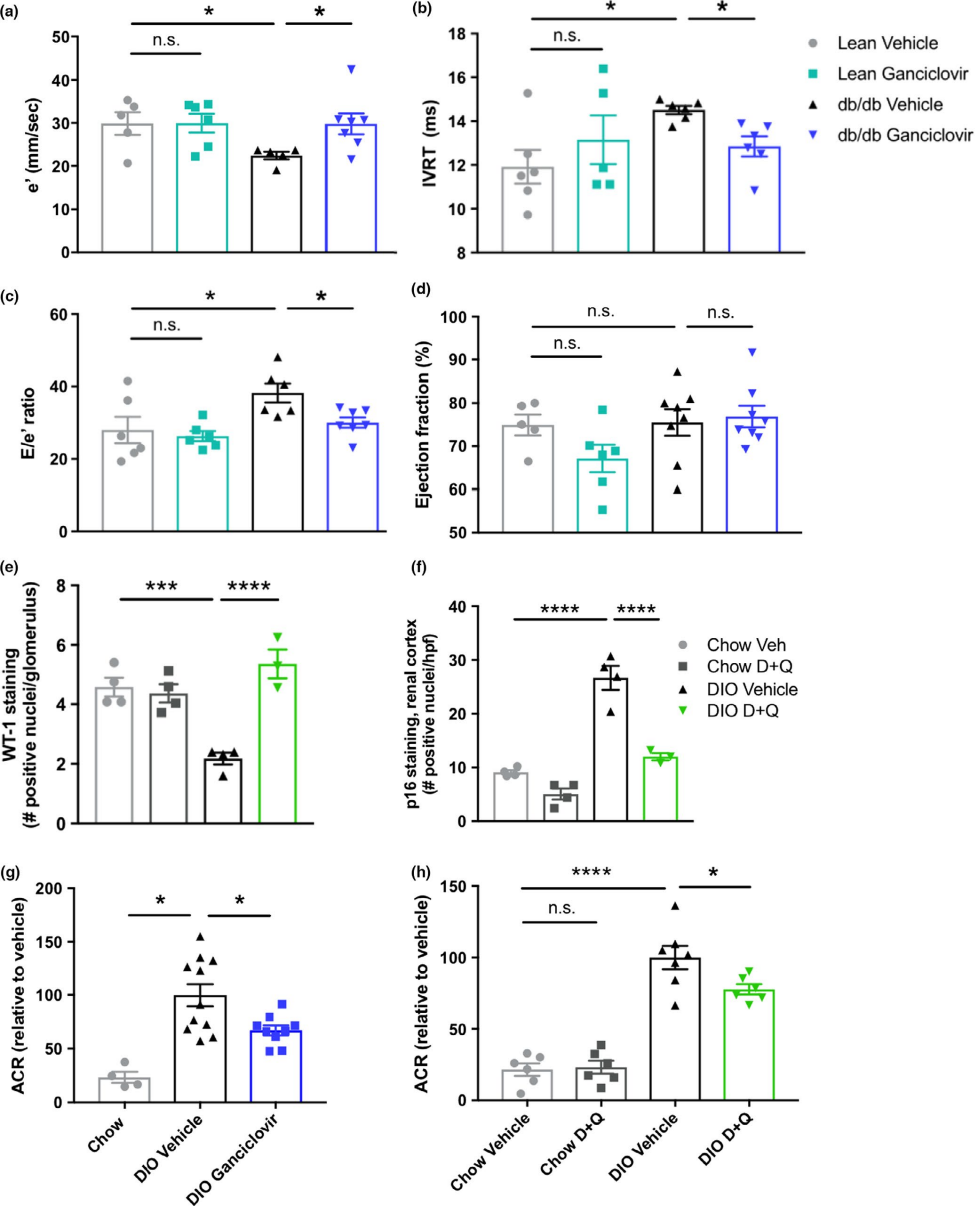
Complications of diabetes are alleviated by senescent cell clearance. (a–c) Parameters of diastolic function in p16‐3MR *db/db* mice treated with ganciclovir determined by echocardiography–Doppler measurements (*n* = 5–8 per group): (a) Early diastolic velocity from medial mitral annulus (e′); (b) isovolumic relaxation time (IVRT) c, left ventricular filling pressure (E/e′) (*n* = 5–8 per group); (d) left ventricular ejection fraction (EF), an indicator of systolic function, in p16‐3MR lean or *db/db* mice treated with ganciclovir (*n* = 5–8 per group). (e) Number of cells per glomerulus staining positively for Wilms tumor protein in D + Q‐treated DIO mice (WT‐1; e, *n* = 4 per group). (f) Average number of p16^Ink4a^‐positive cells per field in the renal cortex of D + Q‐treated DIO mice (*n* = 4 per group). (g, h) Urine albumin/creatinine ratio (ACR) in DIO p16‐3MR mice treated with ganciclovir (g, chow *n* = 4, DIO *n* = 9–11 per group) or DIO wild‐type mice treated with D + Q (h, *n* = 6–9 per group). Means ± *SEM* are shown. *p < 0.05, ***p < 0.0005, ****p < 0.0001; one‐way ANOVA with Bonferroni correction or two‐tailed Student's *t* test when comparing two groups

Because our interventions target senescent cells not only in adipose, but also in other tissues, their potential effects could exceed those limited to alleviating adipose tissue dysfunction. Senolytics may affect senescence‐related comorbidities associated with obesity (e.g., hepatic steatosis, osteoarthritis, and neuropsychiatric dysfunction), accelerated aging‐like states associated with obesity (e.g., sarcopenia and frailty), and the consequences of chronological aging itself by acting on nonadipose senescent cells (Kirkland et al., 2017; Ogrodnik et al., 2019; Palmer et al., 2015). They may also decrease the complications of diabetes and obesity related to persistent inflammation caused by senescent cell accumulation, including cardiovascular and renal dysfunction. Indeed, senescent cell clearance enhanced diastolic function in obese mice (Figure 5a–c; systolic function as measured by left ventricular ejection fraction [EF] was unchanged [Figure 5d]), consistent with earlier reports in other models that senescent cells contribute to cardiac dysfunction, vascular stiffness, and atherosclerosis (Childs et al., 2016; Demaria et al., 2017; Roos et al., 2016; Zhu et al., 2015). Additionally, renal podocytes had increased expression of Wilms tumor protein (Wt‐1), a measure of podocyte integrity and function (Figure 5e; Guo et al., 2002), after D + Q treatment, which also decreased p16^Ink4a+^ cells in renal cortex (Figure 5f). Furthermore, we found decreased urinary albumin to creatinine ratio (ACR), an indicator of renal dysfunction, after targeting senescent cells genetically or with senolytic drugs in obese mice (Figure 5g,h). Thus, senolytics may alleviate senescence‐associated complications of metabolic dysfunction, beyond the impact of glucose‐lowering and insulin sensitization that senolytics share with certain currently available diabetes treatments.

## 3 DISCUSSION

Our study indicates in several different models that senescent cell clearance may be an effective strategy for alleviating important elements of obesity‐related metabolic dysfunction. Senolytic treatment recapitulated metabolic improvements seen after transgene‐mediated clearance of senescent cells. Senescent cells may not only drive insulin resistance directly through their SASP, but also promote adipose tissue immune cell infiltration and impair progenitor cell function. More investigation is needed into the relative contributions of each of these mechanisms to insulin resistance and complications of diabetes.

The transgenic models used in these studies are dependent on targeting high p16^Ink4a^ expression. Not every senescent cell has high p16^Ink4a^, and there are cell types that have a transient elevation in p16^Ink4a^ that are not truly senescent, such as activated macrophages (Hall et al., 2017). These points suggest that interpreting studies based on targeting cells with high expression of p16^Ink4a^ alone requires caution, so we also used senolytics to test our hypothesis. Effects of D + Q do not depend on high p16^Ink4a^ expression, but rather on transiently disabling the survival pathways protecting senescent cells from apoptosis.

It is possible that other senolytic agents may have distinct or complementary effects to those of D + Q in diabetes, since senolytics vary considerably in the spectra of senescent cell types that are targeted. Thus, targeting different types of senescent cells and determining the effects of various combinations of different senolytics, as well as behavioral interventions (e.g., exercise [Schafer et al., 2016] or dietary interventions), will be important next steps in harnessing the benefits of these drugs. This might open opportunities to create individualized treatments by targeting particular senescent cell types in patients with different constellations of symptoms and complications of metabolic disease. Another key step will be to test effects of different senolytics on metabolic dysfunction and its complications across a broad range of animal models of diabetes, including type 1 diabetes, and studies in primates in preparation for clinical trials.

Importantly, initial human trials of senolytics for other conditions are underway. Indeed, the first clinical trial of senolytics was recently reported. Improvements in physical function of patients with idiopathic pulmonary fibrosis were found in a pilot study of D + Q (Justice et al., 2019). Similar pilot clinical trials and ultimately larger, longer duration, randomized double‐blind placebo‐controlled trials are needed to test the potential therapeutic benefits of senolytics in diabetic patients, including alleviation of complications of metabolic disease such as cardiovascular, renal, and neurological dysfunction. Critically, since D + Q was given intermittently and the elimination half‐life of each drug is <12 hr (Christopher et al., 2008; Graefe et al., 2001), the observed effects are consistent with reduced senescent cell burden, rather than effects on senescence‐independent signaling pathways, receptors, or enzymes that require the sustained presence of drugs. Thus, intermittent administration of senolytics may delay or alleviate diabetes, complications of both obesity and diabetes, and other comorbidities associated with senescence‐related chronic diseases, a possibility that, if further confirmed in pre‐clinical studies, merits examination in clinical trials.

## 4 EXPERIMENTAL PROCEDURES

### 4.1 Animals

Mice heterozygous for either the p16‐3MR or INK‐ATTAC transgenes on a C57BL/6 background were used in these experiments (Demaria et al., 2017; Xu, Palmer et al., 2015). J.L.K., T.T., J. van Deursen, and D. Baker (all currently at Mayo) devised the strategy and characterized INK‐ATTAC mice (Baker et al., 2011). The ATTAC construct was devised by P. Scherer, who sent it to J.L.K. p16‐3MR mice were generously provided by Dr. Judith Campisi. Leptin receptor knockout heterozygotic (Lepr^db/−^) mice were purchased from Jackson Laboratory (Bar Harbor, ME) and crossed to either p16‐3MR mice or INK‐ATTAC mice to generate mice homozygous for the leptin receptor mutation (*db/db*). CAG‐luc transgenic C57BL/6 mice were obtained from the Jackson Laboratory (Stock No: 025854). The CAG‐luc mouse expresses firefly luciferase driven by the constitutively active CAG promoter in most tissues. Ages of mice used in each experiment are reflected in Supporting Information Figure S1. Male and female mice were used for *db/db *experiments. Male mice were used for DIO experiments due to their increased susceptibility to high‐fat diet‐induced metabolic dysfunction. For diet‐induced obesity (DIO) experiments, mice were maintained on a 60% (by calorie) fat diet (D12492, irradiated; Research Diets, New Brunswick, NJ). Lean control mice were fed a normal chow diet (LabDiet 5053, 13.2% fat by calorie, St. Louis, MO). Mice were maintained under a 12‐hr light and 12‐hr dark cycle at 24°C with free access to food and water in a pathogen‐free facility. All studies were approved by the Mayo Clinic Institutional Animal Care and Use Committee.

### 4.2 Cell strains

*THP‐1 cells* were purchased from ATCC (Manassas, VA).

*Human adipocyte progenitors*, also termed adipose‐derived stem cells or preadipocytes (Tchkonia et al., 2013), were isolated from abdominal subcutaneous adipose tissue biopsies from nine healthy subjects undergoing surgery to donate a kidney (age 37 ± 6 years, male, BMI 26 ± 2). These cells were used for osteopontin measurements in senescent cells and for THP‐1 migration studies. These studies were approved by the Mayo Clinic Institutional Review Board.

### 4.3 Drug treatments

Ganciclovir (Genentech, San Francisco, CA; 25 mg/kg), dissolved in water and phosphate‐buffered saline [PBS]) or PBS alone, was administered i.p. for five consecutive days, with 10–14 days between the last dose of one treatment round and the first day of the next treatment round. AP20187 (MedchemExpress, Monmouth Junction, NJ; 10mg/kg, dissolved in ethanol for stock solution, then diluted in PEG‐400 and 2% Tween for dosing solution) or PEG‐400/Tween 2% in water (vehicle) was administered by i.p. injection for three consecutive days, with 12 days between treatments. Dasatinib (LC Laboratories, Woburn, MA; 5 mg/kg) and quercetin (Sigma Aldrich, St. Louis, MO; 50 mg/kg) or vehicle (60% Phosal, 10% ethanol, and 30% PEG‐400) were administered for five consecutive days monthly *via* oral gavage in cohort 1, or for three consecutive days, with 14 days between the last dose of one treatment round and the first day of the next treatment round in cohort 2. For CyTOF experiments, a single course of AP20187 (3 days) or D + Q (5 days) was given 3 days prior to sacrifice. Navitoclax (Active Biochem, Kowloon, Hong Kong; 50 mg/kg) or vehicle (60% Phosal, 10% ethanol, and 30% PEG‐400) was administered *via* oral gavage for five consecutive days monthly. Body composition was measured by EchoMRI (Echo Medical Systems, Houston, TX).

### 4.4 Metabolic testing

For DIO and lean mice, glucose tolerance and insulin tolerance testing (ipGTT, ITT, respectively) were performed by injecting 1.2 g/kg glucose or 0.8 mU/kg insulin (i.p.) in the early afternoon following a 6‐hr or 4‐hr fast, respectively. Glucose‐stimulated insulin secretion (GSIS) in DIO and lean mice was measured after oral gavage of 1.5 g/kg glucose following a 4‐hr fast. Intraperitoneal injection of glucose was used for GTT due to increased excursion of blood glucose, allowing for better differentiation between treatment groups. Oral administration of glucose was used for insulin secretion measurements in Figure 2h to ensure the most physiologic induction of insulin secretion (including the contribution of incretins), as well as to test the overall validity of our results by testing improvements with senescent cell clearance in multiple assays. For *db/db* mice, glucose tolerance testing was performed by injecting 1.0 g/kg glucose i.p. following an overnight fast. Tail vein blood glucose was measured at time 0 and at indicated time points using a handheld glucometer (Bayer Breeze 2 [Bayer, Whippany, NJ], or for *db/db *mice, AlphaTrak [Zoetis, Parsippany, NJ]). Aside from clear weight differences between lean and DIO or *db/db* mice, the experimenter conducting GTT, ITT, and GSIS testing was unaware of the treatment group assignment of each mouse during testing. HbA1c was measured using a commercial test (A1cNow+, Bayer, Whippany, NJ). Plasma insulin, adiponectin, activin A, and osteopontin were measured using commercial ELISA kits (insulin and adiponectin: ALPCO, Salem NH; osteopontin and activin A: R&D Systems, Minneapolis MN).

### 4.5 Hyperglycemic clamp experiments

Mice were fasted from 9 hr before the experiment until the end of the experiment. The study was divided into three periods, (a) a basal period in which only glucose tracer was infused, (b) a hyperglycemic period (HGC) in which, next to the glucose tracer, glucose was infused at a variable rate to maintain blood glucose levels at about 20 mM, and (c) a hyperinsulinemic–euglycemic period (HIEC) during which, in addition next to the glucose tracer, insulin was infused at a constant rate and glucose was infused at a variable rate to maintain blood glucose levels at about 6 mM. During all three periods, blood glucose levels and glucose infusion rates were monitored, from which kinetic parameters were calculated.

### 4.6 Adipocyte progenitor isolation

The stromal vascular fraction of perigonadal adipose tissue was isolated by collagenase digestion as previously described (Tchkonia et al., 2005).

### 4.7 Mass cytometry (CyTOF)

We designed a panel of antibodies based on surface markers, transcription factors, and cytokines (see Supporting Information Table S1). Each antibody was tagged with a rare metal isotope and its function verified by mass cytometry according to the factory manual (Multi Metal labeling kits; Fluidigm South San Francisco, CA). A CyTOF‐2 mass cytometer (Fluidigm) was used for data acquisition. Acquired data were normalized based on normalization beads (Ce140, Eu151, Eu153, Ho165, and Lu175). Stromal vascular cells were isolated from epididymal white adipose tissue. Collected cells were incubated with metal‐conjugated antibodies and for testing intracellular proteins, including transcription factors and cytokines. Fixation and permeabilization were conducted according to the manufacturer's instructions (Foxp3/ Transcription Factor Staining Buffer Set, eBioscience, San Diego, CA). CyTOF data were analyzed by Cytobank (Santa Clara, CA). Total progenitors were defined as CD45^−^, CD31^−^, and Sca1^+^. Macrophages were defined as F4/80^+^ and CD11b^+^. Endothelial cells were defined as CD31^+^ and CD146^+^. T lymphocytes were defined as CD4^+^ or CD8^+^. Upon testing of antibodies to *Renilla* luciferase, we found that background signals were too high in WT mice to be specific enough to use in these experiments. Therefore, we pursued CyTOF experiments in the INK‐ATTAC model rather than the p16‐3MR model.

### 4.8 Plasma cytokine profiling

Plasma cytokines were quantified using multiplex ELISA on a Bio‐Plex 200 analyzer by Eve Technologies (Calgary, Alberta, Canada).

### 4.9 Quantitative real‐time PCR

Quantitative real‐time PCR was performed as described previously (Xu, Palmer et al., 2015). Primer catalog numbers are in Supporting Information Table S2.

### 4.10 Western blotting

Western blotting was performed as described previously (Xu, Palmer et al., 2015).

### 4.11 Adipose tissue analyses

SA‐β‐gal activity was assayed as reported previously (Xu, Palmer et al., 2015). Briefly, a small piece of adipose tissue was collected in PBS, lightly fixed with glutaraldehyde and formaldehyde for 15 min, washed 3X in PBS, and placed in SA‐β‐gal activity solution containing X‐gal at pH 6.0 at 37°C for 14–16 hr. Tissues were rinsed in PBS, nuclei were stained with DAPI, and adipose tissue was compressed between two glass slides for light microscopy. SA‐β‐gal^+^ cells as a percent of all nuclei were quantified in at least 10 images taken at random per tissue using NIS‐Elements software (Nikon Instruments, Melville, NY). Neither the person who captured images for quantification nor the observer counting SA‐β‐gal^+^ cells in the captured images was aware of the identity of the samples. Photographs of SA‐β‐gal^+^ tissue chunks were taken after 5–8 hr of incubation at 37°C. Cell size was quantified using at least 10 images taken at random from adipose tissue chunks by light microscopy by measuring diameters of all completely visible adipocytes per field using Nikon NIS‐Elements software.

### 4.12 Ex vivo insulin response assay

Adipose tissue was cut into small pieces (<100 mg) and washed with PBS 3 times. The minced tissue was treated with 5 nM insulin or vehicle (PBS) for 5 min at 37°C. p‐AKT (#4060) and total‐AKT (#4691) antibodies for Western analyses were purchased from Cell Signaling (Danvers, MA).

### 4.13 Albumin–creatinine ratio

Albumin was measured by ELISA (GenWay, San Diego, CA), and creatinine was measured by colorimetric assay (MaxDiscovery; Bio Scientific, Austin, TX) in urine collected from nonfasted mice. Albumin–creatinine ratio (ACR) was calculated by determining microgram of albumin per mg of creatinine.

### 4.14 Conditioned medium experiments

Stromal vascular collagenase digests were plated, replated within 18 hr, and serially sub‐cultured for 4 population doublings using differential plating and culture media designed to select against macrophage or endothelial cell contamination. Cells were exposed to 10 Gy radiation or were sham‐irradiated. After 25 days, irradiated cells acquired senescent morphology, developed SA‐β‐gal activity, and expressed p16^INK4A^ and SASP components. Cells were washed, and serum‐free conditioned medium (CM) was collected and passed through a 0.2‐µm filter. CM was concentrated using 3K concentrators (Amicon Ultra‐15 Centrifugal Filter Units, Millipore, Darmstadt, Germany) and analyzed by LC/MSMS (Q Exactive™ Hybrid Quadrupole‐Orbitrap™ Mass Spectrometer, ThermoFisher, Waltham, MA) for osteopontin.

### 4.15 Adipocyte progenitor differentiation

To induce differentiation of mouse cells, 80% confluent adipocyte progenitors were exposed to differentiation medium containing DMEM/F‐12, 10% FBS, 1μg/ml insulin, 250 nM dexamethasone, 0.5mM IBMX, and 2.5μM rosiglitazone for 48 hours at 20% O_2_. Medium was then replaced with DMEM/12 containing only 10% FBS and insulin, and kept in culture for an additional 5–8 days.

### 4.16 Comprehensive laboratory animal monitoring system

Metabolic rate and food intake were measured using a Comprehensive Laboratory Animal Monitoring System (CLAMS) as previously described (Xu, Tchkonia et al., 2015).

### 4.17 THP‐1 macrophage migration

Migration assays were performed using Transwell polycarbonate membrane inserts with a 5‐μm pore diameter purchased from Corning (Corning, NY). THP‐1 cells were stained with CellTracker CM‐DiI dye (Thermo Fisher Scientific, Waltham, MA) according to the manufacturer's directions and then plated into the inserts. Nonsenescent control or senescent human primary adipocyte progenitors were seeded into the bottom chambers of the plates. After 24 hr, DiI‐labeled THP‐1 cells that had migrated into the bottom chamber were imaged.

For neutralizing antibody experiments, THP‐1 cells (10^5^) were plated into Transwell inserts and allowed to migrate into the bottom chamber for 6 hr at 37°C. After 6 hr, the number of cells that had migrated into the lower chamber was quantified using the CyQUANT Cell Proliferation Assay Kit (Life Technologies) according to the manufacturer's instructions. All neutralizing antibodies were purchased from R&D. The concentrations for the antibodies in the CM are Goat IGG (1 mg/ml), M‐CSF (1 mg/ml), MCP1 (0.2 mg/ml), and MIP‐1b (0.2 mg/ml).

### 4.18 Monocyte infiltration

Monocytes were isolated from femur and tibial bone marrow of CAG‐luc transgenic C57BL/6 mice using a commercial kit (Monocyte Isolation kit, MACS Miltenyi Biotec, San Diego, CA). 1 × 10^6^ monocytes in PBS were injected i.v. *via* the tail vein in *db/db* or lean mice. Luminescent signal was examined 24 hr after monocyte injection.

### 4.19 Luminescence imaging

Both *Renilla* and firefly luminescence imaging were performed using a Xenogen IVIS 200 system (Caliper Life Sciences, Hopkinton, MA). To detect p16‐3MR *Renilla* luciferase‐expressing cells, 200 µl coelenterazine h (Rediject, PerkinElmer) was injected i.p. into each mouse 10 min before imaging was initiated. For firefly luminescence, 3 mg d‐luciferin (Gold Biotechnology, St. Louis, MO) in 200 µl PBS was injected i.p. into each mouse 5 min before imaging was initiated. The exposure for luminescence images was 5 min.

### 4.20 Echocardiography

High‐resolution ultrasound imaging was used to evaluate cardiac function as previously described (Roos et al., 2016).

### 4.21 Anti‐Wilms tumor protein immunohistochemistry

Formalin‐fixed paraffin‐embedded mouse kidney tissue sections were subjected to steam heat‐mediated antigen retrieval for 30 min in 10 mM sodium citrate buffer, pH 6.0. Samples were then incubated at room temperature for 1 hr with primary antibody (Anti‐Wilms Tumor Protein; Abcam #ab89901) diluted 1/100, followed by a 30‐min incubation in a Ready‐to‐Use HRP‐Labelled secondary antibody (Dako K4003). Color was developed using Vector NovaRED Peroxidase Substrate Kit (SK‐4800) followed by hematoxylin counterstaining.

### 4.22 Quantification and statistical analyses

When comparing two groups, unpaired two‐tailed Student's *t* tests were used. *p* < 0.05 was considered significant. One‐way ANOVA with Bonferroni correction was used to estimate statistical significance when more than two groups were being compared. All values are expressed as mean ± *SEM*. Sample sizes were chosen based on the means and variation of preliminary data to achieve at least 80% power and allowing for 5% type I error.

## CONFLICT OF INTEREST

J.L.K., T.T., T.P., A.K.P., Y.Z., M.X., J.C., and M.D. have a financial interest related to this research. Patents on senolytic drugs are held by Mayo Clinic. This research has been reviewed by the Mayo Clinic and Buck Institute Conflict of Interest Review Boards and was conducted in compliance with Mayo Clinic and Buck Institute Conflict of Interest policies. None of the other authors has a relevant conflict of financial interest.

## AUTHOR CONTRIBUTIONS

A.K.P., J.L.K., M.X., Y.Z., and T.T. conceptualized the study and designed the methodology. A.K.P., M.X., Y.Z., M.M.W., C.M.H., M.O., T.P., T.H.D., E.V., T.W., G.C‐V., K.O.J., H.C., M.B.S., J.G., and T.T. performed the experiments and conducted analyses. A.K.P., M.X., Y.Z., T.T., and J.L.K. wrote the manuscript. All authors discussed the results and commented on the manuscript. J.L.K. provided overall supervision.
