# Supplementary material for: Targeting senescent cells alleviates obesity‐induced metabolic dysfunction
Source: Aging Cell. 2019 Mar 25;18(3):e12950. doi: 10.1111/acel.12950 (PMC6516193; doi:10.1111/acel.12950)
Supplement: Supplementary file 7 [file ACEL-18-e12950-s007.docx]

**Supplementary Table 1. Antibodies used for mass cytometry**

| **Targets** | **Clone** | **Vendor** | **Cat#** | **Metal Isotopes** | **Dilution** |
| --- | --- | --- | --- | --- | --- |
| p21 | F-5 | Santa Cruz | sc-6246 | 144Nd | 1/50 |
| DYKDDDDK FLAG | L5 | Biolegend | 651501 | 170Er | 1/50 |
| CENP-B | Rabbit polyclonal | Abcam | ab25734 | 145Nd | 1/100 |
| F4/80 | BM8 | Fuidigm | 3146008B | 146Nd | 1/200 |
| CD11b (Mac-1) | M1/70 | Fuidigm | 3154006B | 154Sm | 1/200 |
| Ki-67 | SolA15 | ebioscience | 14-5698-82 | 162Dy | 1/400 |
| CD45 | 30-F11 | Fuidigm | 3089005B | 89Y | 1/400 |
| Ly-6A/E (Sca-1) | D7 | Fuidigm | 3164005B | 164Dy | 1/400 |
| CD31 (PECAM-1) | 390 | Fuidigm | 3165013B | 165Ho | 1/200 |
| TNFα | MP6-XT22 | Biolegend | 506302 | 176Yb | 1/100 |
| CD146 | ME-9F1 | Fuidigm | 3141016B | 141Pr | 1/200 |
| CD117 (ckit) | 2B8 | Fuidigm | 3166004B | 166Er | 1/50 |
| CD4 | RM4-5 | Fuidigm | 3172003B | 172Yb | 1/200 |
| CD8a | 53-6.7 | Fuidigm | 3153012B | 153Eu | 1/201 |
| CD3e | 145-2C11 | Fuidigm | 3165020B | 152Sm | 1/202 |
| CD29 | eBioHMb1-1 | ebioscience | 14-0291-82 | 161Dy | 1/400 |
| CD34 | RAM34 | ebioscience | 14-0341-82 | 147Sm | 1/200 |

**Supplementary Table 2. Oligonucleotides**

| **Oligonucleotide** | **Source** | **Identifier** |
| --- | --- | --- |
| Cdkn2a / p16^Ink4a^ | IDT | Mm.PT.58.42804808 |
| Pparγ | ThermoFisher | Mm00440940_m1 |
| Cebpα | ThermoFisher | Mm00514283_s1 |
| Fapb4 | ThermoFisher | Mm00445878_m1 |
| Adipoq (Adiponectin) | ThermoFisher | Mm00456425_m1 |
| Adgre1 (F4/80) | ThermoFisher | Mm00802529_m1 |
| Spp1 | ThermoFisher | MM00436767_m1 |
| Tbp | ThermoFisher | Mm00446971_m1 |
| Luciferase  Primers:  FW: 5'-GACGTGCCTCCACAGGTAG -3'  REV: 5'-CGAGAACGCCGTGATTTT-3' | Roche (Indianapolis, IN) | UPL Probe #16 |
| mRFP  Primers:  FW 5’ -GACCTCGGCGTCGTAGTG–3’  RV 5’–AAGGGCGAGATCAAGATGAG–3’ | Roche | UPL Probe #161 |
| eGFP  Primers:  FW: 5’-CCCATACAATGGGGTACCTTC-3’  REV: 5’-TGGCTCTCCTCAAGCGTATT-3’ | Roche | UPL Probe #41 |
